# Voronoi Tessellations and the Shannon Entropy of the Pentagonal Tilings

**DOI:** 10.3390/e25010092

**Published:** 2023-01-02

**Authors:** Edward Bormashenko, Irina Legchenkova, Mark Frenkel, Nir Shvalb, Shraga Shoval

**Affiliations:** 1 Chemical Engineering Department, Engineering Faculty, Ariel University, P.O.B. 3, Ariel 407000, Israel; 2 Department of Mechanical Engineering & Mechatronics, Faculty of Engineering, Ariel University, P.O.B. 3, Ariel 407000, Israel; 3 Department of Industrial Engineering and Management, Faculty of Engineering, Ariel University, P.O.B. 3, Ariel 407000, Israel

**Keywords:** Shannon entropy, pentagonal tiling, Marjorie Rice Tiling, Voronoi tessellation, iso-symmetrical transitions

## Abstract

We used the complete set of convex pentagons to enable filing the plane without any overlaps or gaps (including the Marjorie Rice tiles) as generators of Voronoi tessellations. Shannon entropy of the tessellations was calculated. Some of the basic mosaics are flexible and give rise to a diversity of Voronoi tessellations. The Shannon entropy of these tessellations varied in a broad range. Voronoi tessellation, emerging from the basic pentagonal tiling built from hexagons only, was revealed (the Shannon entropy of this tiling is zero). Decagons and hendecagon did not appear in the studied Voronoi diagrams. The most abundant Voronoi tessellations are built from three different kinds of polygons. The most widespread is the combination of pentagons, hexagons, and heptagons. The most abundant polygons are pentagons and hexagons. No Voronoi tiling built only of pentagons was registered. Flexible basic pentagonal mosaics give rise to a diversity of Voronoi tessellations, which are characterized by the same symmetry group. However, the coordination number of the vertices is variable. These Voronoi tessellations may be useful for the interpretation of the iso-symmetrical phase transitions.

## 1. Introduction

The research of families of pentagons admitting tilings (covering) of the plane has a rich and intriguing history. The first five types which admit tile-transitive tessellation of the plane were suggested by Reinhardt [1]. Reinhardt was an assistant of David Hilbert. Hilbert’s 18th problem asks whether or not there exist 3-dimensional tiles that admit only non-tile-transitive tessellations. It is customary to think that David Hilbert did not state the 2-dimensional version of this problem as he believed that no such polygons exist [2]. Reinhardt solved Hilbert’s 18th problem by demonstrating a 3-dimensional tile that admits only non-tile-transitive tilings of 3D space [2]. However in the same article, Reinhardt asserted (but did not prove) that a 2D analog does not exist. Heesch demonstrated a counterexample to Reinhardt’s suggestion by demonstrating a 2D tile that admits only non-tile transitive tessellation, thus introducing convex pentagons admitting only non-tile-transitive tilings [2,3]. Afterwards, Reinhardt himself discovered five pentagonal tessellations of the plane. Additional convex pentagons that admit only non-tile transitive tilings were suggested by Kershner [2,4]. The set of pentagons enabling the tile-transitive tessellation of the plane was discovered by Marjorie Rice, an amateur mathematician and mother of five, who had become a follower of Martin Gardner’s long-running column, “Mathematical Games”I published in the Scientific American magazine [5,6].

The computer classification the convex pentagons that admit paving of the plane was suggested in Ref. [2]. An exhaustive search of convex pentagons which tile the plane was reported in Ref. [7]. M. Rao demonstrated that that there are no more than the already 15 known families [7]. We call these families further in the text “basic pentagon tiles”, or for sake of brevity “basic tiles” or “basic pentagons”.

It seems that the pentagonal tilings resemble the famous Penrose tiling, built from pairs of shapes and demonstrating the 5-fold rotational symmetry [8]. However, the pentagonal tilings and Penrose tilings are actually very different: the Penrose tiling is an example of an aperiodic tiling [9]. In other words, the Penrose tiling represents covering of the plane by non-overlapping polygons, in which shifting any tiling with these shapes by any finite distance, without rotation, cannot produce the same tessellation. The translational symmetry is absent in the Penrose tiling. Contrastingly, the pentagonal tilings, addressed in our paper, are periodic tessellations, characterized by the translational symmetry. Thus, the Penrose and pentagonal tiling represent very different classes of mathematical objects.

Penrose tiling was extremely useful for explanation of the structure of quasi-crystals [10,11,12]. The very question is: what is the physical exemplification of the pentagonal tiling? We demonstrate that the pentagonal tiling may be used for the explanation of the iso-symmetric phase transitions, which were discovered recently [13,14,15,16]. The method used in our investigation is based on the building of the Voronoi diagrams on the set of points emerging from the pentagonal tiling. Vertices of the pentagons are seen as the seed/nuclei points, generating the coressponding Voronoi diagrams (Voronoi tessellations).

Partitioning of an infinite plane into regions based on the distance to a specified discrete set of points (called seeds or *nuclei*) constructs the Voronoi tessellation. There is a corresponding region for each seed, consisting of all points closer to that seed point (also called generators) than to any other point [17,18,19,20,21,22]. The Voronoi diagram of the addressed Penrose tiling was composed of *N* polygons. For any given set of points corresponding to the Voronoi tessellation or diagram, the Shannon/Voronoi entropy is defined by Equation (1):

(1) $$S=-\sum P_{n}lnP_{n}$$

where *P_n_* is the fraction of polygons with *n* sides or edges (also called the coordination number of the polygon) in a given Voronoi diagram [10,11,12]. In our paper, we analyzed Voronoi tessellations emerging from the 15 known pentagonal tilings. Recently, we investigated the Voronoi diagrams generated by the Penrose tiling, and demonstrated fruitfulness of such an analysis [23,24]. The study of the Voronoi diagrams emerging from the pentagonal tilings also supplied non-trivial results.

## 2. Methods

**Figure 1:**
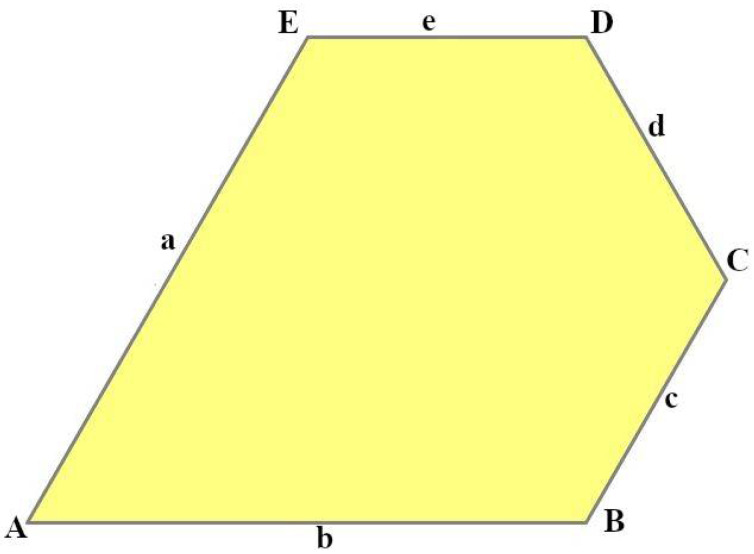
Pentagonal basic tile example with vertices A, B, C, D, E and edges a, b, c, d, and e.

We studied 15 pentagonal tilings arising from 15 types of basic pentagons, as they were classified in Ref. [7]. Figure 1 depicts a pentagonal tile; the edges of the tile are labeled *abcde*, the angles are labeled *ABCDE*.

The basic pentagons, as they were classified in Ref. [7], are described in Table A1. The majority of these classes allows change in their shape, under variation of their edges and angles; in particular, it is possible to change edges and angles of the classes labeled 1, 2, 3, 4, 5, 6, 7, 8, 9, 10, 11, 12 and 13. These tilings are called in the text “flexible tessellations”. Visualization of the change in the shape of the flexible tilings was carried out with the “Tiling Viewer” software by Jaap Scherphuis, which can be found on the following link https://www.jaapsch.net/tilings/applet.htm (accessed on 1 March 2012). An example of such a transformation of Tile 11 is shown in Table 1.

We used 15 tessellations built of pentagons as generators of the Voronoi diagrams (see Column Tiling in Table A1). All of the studied pentagon tilings are classified and depicted in Appendix A (Table A1). Vertices of the pentagons were used as “seeds” for the generation of Voronoi tessellations. One of the problems inherently arising when the 2D tessellations are studied is the problem of uneven edges of the 2D sample. We tried to obtain a tiled area with the most uniform edges. Therefore, the studied tilings comprised a different number of tiles, namely parquets comprising from 100 to 375 tiles. Then the vertices of the pentagons were used as the “seeds” for the generation of the Voronoi tessellations. This procedure was performed for every of the studied pentagonal tiling.

Flexible tilings generated a variety of Voronoi diagrams, shown in Table 1 and Table A1 (See Appendix A). It should be emphasized that the source/basic pentagon-based tilings gave rise to tessellations built of several (from 1 to 5) kinds of polygons to be discussed below. However, no Voronoi tiling built only of pentagons was registered.

## 3. Results

### 3.1. Voronoi Diagrams Generated by the Marjorie Rice Tiling 11

Let us exemplify the suggested approach with the analysis of the Voronoi diagrams emerging from the Marjorie Rice Tiling 11 (see Table 1). This tessellation is flexible, and it gives rise to a variety of basic tessellations and correspondingly to the Voronoi diagrams arising from these tessellations.

Let us explain data supplied in Table 1. Row 1 supplies the general data related to the basic Marjorie Rice Tiling 11. Small letters (*a*, *b*, *c*) denote edges of the tiling; capitals (*A*, *B*, *C*) denote angles of the tiling. Row 2 of the table depicts the modifications of Tiling 11; row 3 supplies the values of edges and angles for various modifications of Tiling 11. Row 4 depicts the corresponding pentagons, constituting Tiling 11. Row 5 depicts the Voronoi diagrams generated by the vertices of various modifications of Tiling 11. Row 6 supplies the values of the Shannon entropy and parameter ζ defined by Equation (2). The types of polygons appearing in the Voronoi diagram are also presented in row 6. Angles, denoted *B*, *C*, D, and *E*, inherent for Tiling 11, enable variation within [140°;158°], [80°;44°], [130°;112°], [100°;136°], respectively. Edges *a, b,* and *c* also enable variation in the flexible Marjorie Rice Tilling 11.

The properties of the Voronoi diagrams were quantified with the Shannon entropy calculated with Equation (1). We also introduced parameter *ζ* which quantifies the ratio of the number of polygon types appearing in the tiling, defined with Equation (2), as follows:

(2) $$\zeta =N_{k}:N_{l}\ldots :N_{z},\;k<l<\ldots <z$$

where $N_{k},N_{l}$, and $N_{z}$ is the number of *k*, *l*, and *z*-edged polygons in an elementary cell of a given Voronoi tiling, *k* and *z* are the minimal and maximal number of polygon edges appearing in a given Voronoi tessellation correspondingly.

### 3.2. Analysis of the Voronoi Diagrams Emerging from the Entire Set of Basic Pentagons

The entire list of the explored source/basic tilings built of pentagons are supplied in Appendix A (Table A1). Vertices of the basic tiles were taken as the seeds of the Voronoi diagrams depicted and characterized in Table 1 and Table A1. The values of the Shannon entropy and ratio *ζ* for the set of 15 tessellations distinguished by Rao in Ref. [7] are supplied in Table 2. It is seen from the data supplied in Table 2 that various Voronoi diagrams are characterized by the same values of the Shannon entropy. Thus, Shannon entropies of different Voronoi tessellations may coincide, and this is true even for Voronoi diagrams emerging from various source tessellations.

Obviously, the Shannon entropy of the source tessellation built of pentagons only is zero. It is recognized that the Shannon entropy of the Voronoi tessellations is varied in a broad range for the mosaics emerging from the same source pentagonal tiling, namely Equation (3) is true for Tiling 11:

0.562 < *S* < 1.368 (3)

By comparison, for a fully random 2D distribution of points (i.e., with a uniform probability distribution of seed points on a plane), the value of S=1.71 has been reported [21,24,25].

Consider that for the plane tiling with the tiles labeled “7–15” the pairs of a pristine pentagon and its mirror reflection were used (see Table 1A). These basic tiles may be considered as single-polygon ones, if the pristine pentagon and its mirror reflection are taken as identical polygons. Some of the polygons are not flexible; for example, the Rolf Stein tile 14 and Mann/McLoud/Von Derau tile 15 (see Table 1A) are rigid and do not enable variation of the geometrical parameters of the pristine pentagons. Thus, they consequently generate a single type of the Voronoi tessellation (see Table 1A). It is noteworthy that the ninth type of tile generated from Marjorie Rice (Tiling 9, see Table 1a) also generates a single type of Voronoi tessellation, and this in spite of the fact that this type of tile is flexible and enables a change in the shape of pristine pentagons. Basic tile 2 generates the maximal variability of the polygons constituting the Voronoi tessellation (see Table 2).

**Figure 2:**
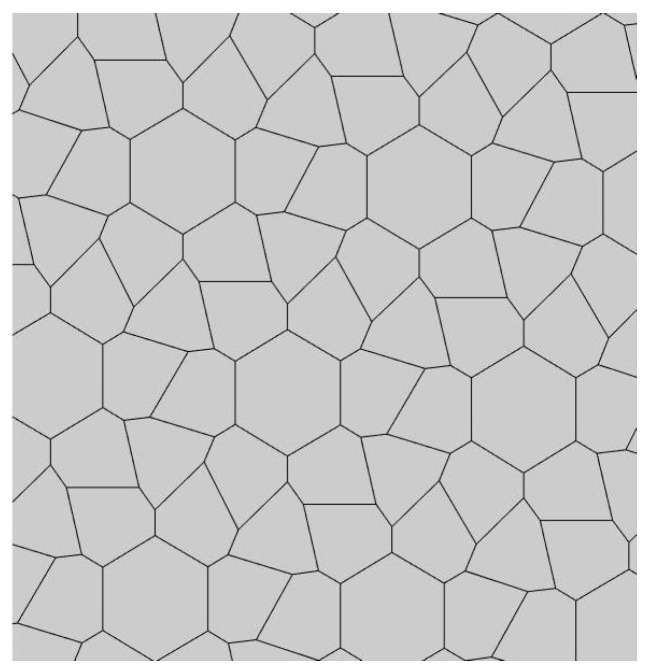
The Voronoi tessellation demonstrating zero Shannon entropy. The tile is generated by the pristine tiling ”5” and it is built of hexagons only.

It was established that 15 basic pristine pentagons generate Voronoi diagrams built of eight types of polygons: from triangles to dodecagons. Somewhat surprisingly, decagons and hendecagon do not appear in the Voronoi diagrams. The most astonishing Voronoi tessellation is depicted in Figure 2. This tiling, emerging from the basic pentagonal tile of the fifth type, is built from hexagons only.

**Figure 3:**
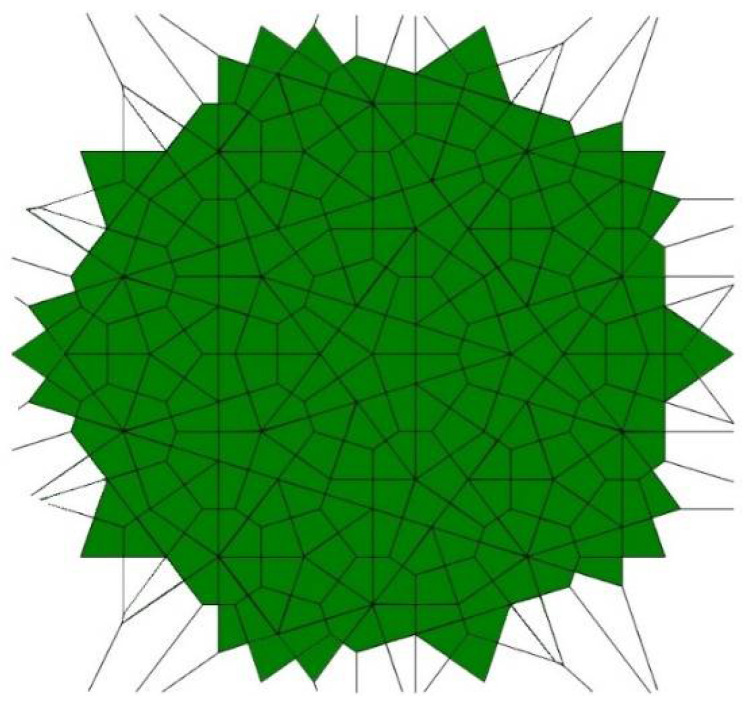
The Voronoi tessellation generated by the Penrose P3 tiling. The centers of the edges of Penrose P3 rhombs are taken as the seeds (*nuclei)* of the Voronoi diagram (for details see Ref. [23]).

Compare the tiling depicted in Figure 2 with that built exceptionally of quadrangles, recently reported in Ref. [23] and shown in Figure 3. The tiling depicted in Figure 3 emerges from the Penrose P3 tiling, in which the centers of the edges of Penrose P3 rhombs are taken as the seeds (*nuclei*) of the corresponding Voronoi diagram.

For both of the tessellations shown in Figure 2 and Figure 3, *S* = 0. Both of these tessellations are built from a mix of regular (symmetric) and irregular (non-symmetric) shapes. The Shannon entropy, which is usually assumed as a measure of “ordering” in the pattern of the tiling depicted in Figure 2, is zero. However, it could hardly be agreed that the pattern shown in Figure 2 is strictly ordered. This example supports the idea that the notion of “ordering” has a fine structure and could not be quantified with a single numerical parameter, such as its Voronoi entropy, as already discussed in Refs. [23,24,26].

**Figure 4:**
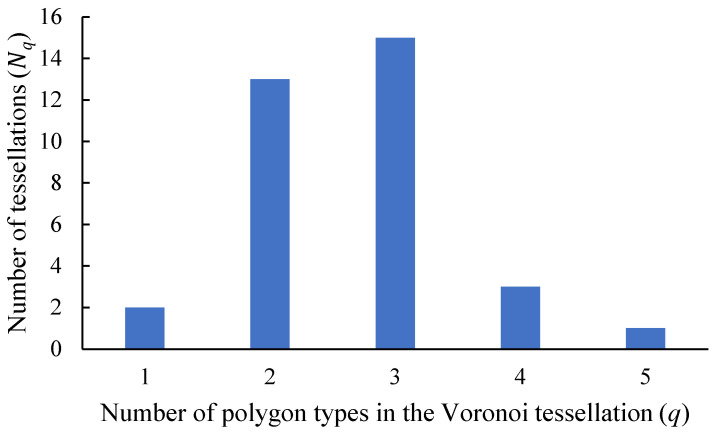
The plot representing the number of Voronoi tessellations *N* built of *q* different types of polygons, denoted $K_{q}$ as a function of number of types of polygons *q* present in the Voronoi tessellation.

**Figure 5:**
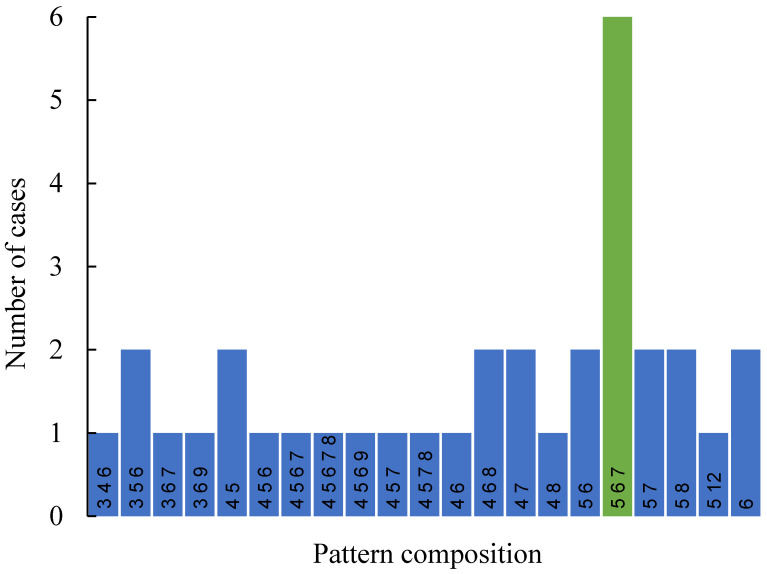
Abundance of appearance of various combinations of polygon types within the entire set of investigated Voronoi tessellations. The triad (346) appearing within a column denotes the tessellation built of triangles, quadrangles and hexagons. The number of occurrences of a given combination of polygons is put at the ordinate axis. The most abundant is the (567) combination, i.e., the Voronoi tessellation built of pentagons, hexagons and heptagons, depicted with the green column.

It was instructive to study the distribution of polygons in the Voronoi diagrams generated by the basic pentagonal tilings of the plane, summarized in Table A1. As already mentioned, the studied Voronoi diagrams may be built from the single type of polygons (hexagons), as shown in Figure 2. However, such mono-tilings were rare in occurrence in our investigation. The most abundant tessellations are built from three kinds of polygons (for example, pentagons, hexagons and heptagons), as shown in Figure 4 and Figure 5. The prevalent polygons are pentagons and hexagons. It should be stressed that no tiling built only of pentagons was registered in our study. It is noteworthy that for the random distribution of seeds the average number of edges surrounding a cell is six in the limit of a large system (provided the averaged coordination number of vertices δ=3), which is an immediate consequence of Euler’s equation in two dimensions, defining the topology characteristics of the surface [19].

Consider the set of Voronoi tessellations arising from basic pristine pentagons and summarized in Table 1A. Let us introduce the parameter *K_q_*, describing the number of Voronoi tessellations *K* built of *q* different types of polygons. Actually, parameter *q* quantifies the variability of polygons within the given Voronoi diagram; the minimal variability of polygons is q=1, which holds for diagrams built of a single type of polygons. In turn, the maximal variability of polygons is q=5, which means that the diagram is built of five types of polygons (the type of a polygon is defined unequivocally by the number of its edges). For example, $K_{q}=13_{2}$ means that that in the entire set of Voronoi tessellations, shown in Table 1A, there appear 13 tessellations built of two different types of polygons (for example pentagons and hexagons). The plot depicting the dependence of $K_{q}$ on the variability *q* of polygons is supplied in Figure 4.

Now let us establish the abundancy of co-occurrence (co-presence) of various polygons within a set of 15 tiles. Various combinations of polygons are present in the investigated Voronoi tessellations, as shown in Figure 5. The notation (3,4,6) means that a given Voronoi tessellation is built of polygons with 3, 4, and 6 vertices (triangles, quadrangles and hexagons). Figure 5 presents the plot of the number of replication of combinations of polygons appearing in the entire set of Voronoi tessellations emerging from 15 types of pentagonal tiling. It is recognized from Figure 5 that the most abundant is the combination of pentagons, hexagons, and heptagons, denoted (5,6,7) and depicted with a green column in Figure 5.

**Figure 6:**
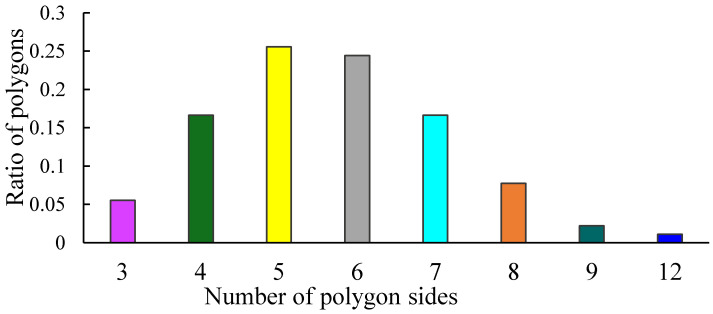
Ratio of polygon types (number of polygon sides n=1, 2…12) appearing within the set of Voronoi tessellations generated by the basic tiles. The most abundant are pentagons and hexagons.

The most abundant within the set of Voronoi tessellations generated by 15 basic tiles are pentagons and hexagons, as shown in Figure 6.

Consider now basic tiles № 3, 5, 6, 7, 8, 10, 11, 13. The surprising properties of Voronoi tessellations generated by these basic tiles were revealed. A variety of Voronoi diagrams are generated by the aforementioned basic tiles. However, for all of the Voronoi diagrams, Equation (4) holds:

(4) $$\overset{i=z}{\underset{i=k}{\sum }}N_{i}=const$$

where $N_{i}$ is the number of *i*-edged polygons in an elementary cell of a given Voronoi tiling, *k* and *z* are the minimal and maximal number of polygon edges appearing in a given Voronoi tessellation correspondingly (see Equation (2)).

## 4. Discussion: Pentagon Tilings, Voronoi Diagrams and Isosymmetric Phase Transitions

### 4.1. Pentagon Tiling and Physics

Let us address the following questions: Why are the Voronoi diagrams emerging from pentagon tilings important? Or, perhaps, are these diagrams of a pure mathematical interest? It should be emphasized that the set of fifteen tile-transitive basic tiles summarized in Table 1A suggests a new kind of spatial ordering of atoms, which may appear in crystals. Moreover, this type of ordering is essentially different from that inherent for quasi-crystals, where translational symmetry is absent [10,11,12]. In contrast, all of the basic pentagonal tiles supplied in Table 1 are characterized by the translational symmetry. However, why are the Voronoi mosaics generated by the basic tiles important for physicists? It should be emphasized that Voronoi tessellations of both rigid and flexible basic tiles remain the symmetry of the pristine tiling untouched, as recognized in Table A1. However, flexible basic tiles generate plane Voronoi patterns in which the location of vertices is different while keeping the symmetry group of the pattern unaltered (see Table A1). At the same time, a change in the Voronoi diagram, emerging from the deformation of flexible source pentagons, points to the change of the coordination number of atoms. Such a change in the geometry of the pattern corresponds to the iso-symmetrical phase transitions, which were recently discovered [13,14,15,16,27]. Phase transitions which have phases of the same space group symmetry are known are iso-symmetric and are necessarily first order. Isosymmetric transitions and/or crossovers occur in important mineralogical systems (pyroxenes, feldspars, and carbonates) and non-linear optic materials (KTiOPO_4_) [13,14,15,16,27].

### 4.2. Pentagon Tiling and Optimal Packing Problems

The set of addressed pentagon tessellations generates a diversity of optimal packing problems. Usually, in these problems, maximal packaging density is calculated [28,29]. In our research, the close-packed tessellations are investigated. Thus, the density of packing by pentagons is maximal for all reported tilings. However, the very interesting problem is formulated as follows: what pentagonal packing (see Table A1) of the circles provides the maximal density when the centers of the circles are located in the vertices of the pentagons? We plan to address this problem in our future investigations. Other optimization parameters may be considered. For example, the following optimal packing problem may be posed: What kind of tessellation provides the optimal ordering within the pattern? It turns out that an answer to this question has a fine structure, due to the fact that ordering could not be unequivocally quantified with a single parameter, as demonstrated in Refs. [23,24]. Shannon entropy, used in the literature for quantifying of ordering, equals zero for of all of the pristine tilings. It also equals zero for the Voronoi tessellation generated by the pristine tiling ”5”, which is depicted in Figure 2 and is built of hexagons only. Obviously the pattern shown in Figure 2 could not be recognized as “strictly ordered”. Thus, other measures quantifying ordering should be considered, such as continuous measure of symmetry, as discussed in Refs. [23,24]. We plan to consider these measures in our future investigations. One more very interesting optimal packing should be mentioned: What kind of tessellations provide the minimal total perimeter of pentagons when an area of the pentagon is fixed? It was recently proved that a regular hexagonal grid or honeycomb has the least total perimeter of any subdivision of the plane into regions of equal area [30]. As to the pentagonal tessellations, it was demonstrated recently that pentagonal Tilings 1 and 2 (see Table 1A) minimize the perimeter among unit area tilings by convex polygons with at most five sides [31].

The perimeter of pentagons also appears in the so-called shape factor ζ, introduced for the quantification of fluid-to-crystal transition in 2D patterns, hidden pattern detection, and the quantification of ordering in colloidal and porous systems, discussed in Refs. [32,33,34,35] and defined according to Equation (5):

(5) $$\zeta =\frac{C^{2}}{4\pi A}$$

where *A* is the surface area and *C* the perimeter of the Voronoi cell correspondingly. For circles ζ=1 and ζ>1 for all other shapes [32,33,34,35]. Flexible tilings labeled 1, 2, 3, 4, 5, 6, 7, 8, 9, 10, 11, 12, and 13 in Table A1 enable the continuous change of ζ to be addressed in our future investigations.

## 5. Conclusions

We investigated properties of the Voronoi tessellations generated by pentagons admitting covering of the plane without gaps and overlapping. The vertices of the basic pentagons were taken as the seeds, generating the Voronoi diagrams. The entire set of these basic polygons includes fifteen types of pentagons, including those introduced by Marjorie Rice. These pentagons are tile-transitive. Some of these pentagons are flexible, i.e., they enable variation of the geometrical parameters: edges and angles. Flexible basic mosaics give rise to a diversity of Voronoi tessellations. The Shannon entropy of these Voronoi tessellations varied in a broad range. For the mosaics emerging from the same source pentagonal tiling (i.e., Tiling 11 supplied in Table A1), we calculated $0.562<S_{vor}<1.368$. Somewhat surprisingly, the Voronoi tessellation emerging from the basic pentagonal tile of the fifth type (see Table A1 and Figure 2) is built from hexagons only. Consequently, the Shannon entropy calculated for this tiling is zero. Decagons and hendecagons did not appear in the investigated Voronoi diagrams. The most abundant Voronoi tessellations are built from three kinds of polygons (pentagons, hexagons, and heptagons). The most widespread is the combination of pentagons, hexagons, and heptagons. The most abundant polygons are pentagons and hexagons. It should be stressed that no Voronoi tiling built only of pentagons was registered in our study. Both basic mosaics and the Voronoi tessellations emerging from basic mosaics are tile-transitive. Thus, in principle, they may describe the planar location of atoms in crystals. Voronoi tessellations keep the symmetry of basic mosaics. However, the coordination number of vertices may be changed for the flexible patterns. Flexible basic pentagonal mosaics give rise to a diversity of Voronoi tessellations which are characterized by the same symmetry group. Thus, these Voronoi tessellations may be useful for the interpretation of the iso-symmetrical phase transitions.

## Figures and Tables

**Table 1:** Flexible Basic Tile 11 gives rise to various Voronoi tessellations. The values of the Shannon entropy and ratio ζ defined by Equation (2) are supplied. Color mapping of Voronoi tessellation in row 5 is carried out as follows: green—quadrangles, yellow—pentagons, gray—hexagons, blue—heptagons, orange—octagons, teal—nonagons.

1	Type 11: 2a + c = d = e; A = 90°; 2B + C = 360°; C + E = 180°; (2D + E = 360°)		
2	** 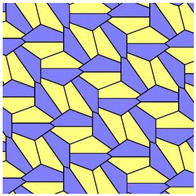 **	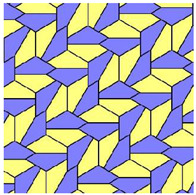	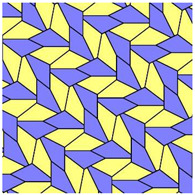
3	A = 90°; B = 145°; C = 70°; D = 125°; E = 110°; a = 28.125, b = 116.55; c = 18.75; d = 75; e = 75	A = 90°; B = 150°; C = 60°; D = 120°; E = 120°; a = 18.75, b = 97.428; c = 37.5; d = 75; e = 75	A = 90°; B = 153.625°; C = 52.75°; D = 116.375°; E = 127.25°; a = 11.25, b = 79.86; c = 52.5; d = 75; e = 75
4	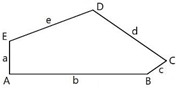	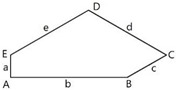	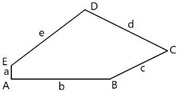
5	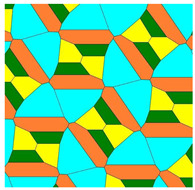	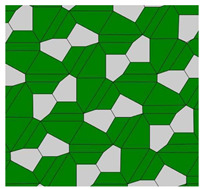	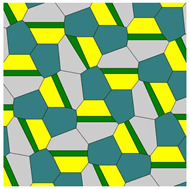
6	4 polygon types: 4, 5, 7, 8-vertices ζ = (1:1:1:1) *S* = 1.386	2 polygon types: 4, 6-vertices ζ = (3:1) *S* = 0.562	4 polygon types: 4 5 6 9-vertices ζ = (1:1:1:1) *S* = 1.386

**Table 2:** Quantitative parameters of the set of Voronoi tessellations emerging from the 15 basic pentagons. The types of basic pentagons are supplied in Table A1. The values the Shannon entropy and parameter *ζ* defined by Equation (2) and quantifying the ratio of polygon types which are present in the given tessellation are supplied.

Pentagon Type	Voronoi Tessellation Parameters, *S* and Ratio *ζ*			
1	2 polygon types: 4 5 *ζ* = 1:1 *S =* 0.693	2 polygon types: 5 6 *ζ* = 1:1 *S* = 0.693	2 polygon types: 5 8 *ζ* = 2:1 *S* = 0.637	
2	2 polygon types: 5 6 *ζ* = 1:1 *S* = 0.693	2 polygon types: 4 7 *ζ* = 1:2 *S* = 0.637	2 polygon types: 4 8 *ζ* = 1:1 *S* = 0.693	2 polygon types: 5 7 *ζ* = 1:1 *S* = 0.693
3	3 polygon types: 3 4 6 *ζ* = 2:3:1 *S* = 1.01	3 polygon types: 3 6 9 *ζ* = 1:4:1 *S* = 0.868		
4	2 polygon types: 4 7 *ζ* = 1:2 *S* = 0.637	3 polygon types: 4 6 8 *ζ* = 1:4:1 *S* = 0.868		
5	3 polygon types: 3 6 7 *ζ* = 2:1:6 *S* = 0.849	3 polygon types: 3 5 6 *ζ* = 2:6:1 *S* = 0.849	1 type: 6 *ζ* = 1 *S* = 0	
6	3 polygon types: 5 6 7 *ζ* = 1:1:1 *S* =1.099	3 polygon types: 3 5 6 *ζ* = 1:1:1 *S* =1.099		
7	2 polygon types: 5 8 *ζ* = 1:2 *S* = 0.637	3 polygon types: 5 6 7 *ζ* = 1:1 *S* = 1.099		
8a	2 polygon types: 4 5 *ζ* = 1:2 *S* = 0.637	3 polygon types: 5 6 7 *ζ* = 1:1:1 *S* = 1.099		
9	3 polygon types: 5 6 7 *ζ* = 1:1:1 *S* =1.099			
10	5 polygon types: 4 5 6 7 8 *ζ* = 2:2:2:4:1 *S* = 1.516	2 polygon types: 5 12 *ζ* = 10:1 *S* = 0.305		
11	4 polygon types: 4 5 7 8 *ζ* = 1:1:1:1 *S* = 1.386	4 polygon types: 4 5 6 9 *ζ* = 1:1:1:1 *S* = 1.386	2 polygon types: 4 6 *ζ* = 3:1 *S* = 0.562	
12	2 polygon types: 5 7 *ζ* = 1:1 *S* = 0.693	3 polygon types: 4 6 8 *ζ* = 1:3:1 *S* = 0.950	4 polygon types: 4 5 6 7 *ζ* = 1:1:1:1 *S* = 1.386	
13	3 polygon types: 5 6 7 *ζ* = 2:3:2 *S* = 1.079	1 type: 6 *ζ* = 1 *S* = 0	3 polygon types: 4 5 7 *ζ* = 1:4:2 *S* = 0.956	
14	3 polygon types: 5 6 7 *ζ* = 1:4:1 *S* = 0.868			
15	3 polygon types: 4 5 6 *ζ* = 1:2:2 *S* = 1.055			

## Data Availability

Not applicable.

## Appendix A

**Table A1:** 15 types of basic tiles (column Tilling) and corresponding Voronoi tessellations generated by the basic tiles (columns Voronoi tessellations). The Voronoi entropy of the patterns and the values of parameter *ζ*, defined by Equation (2) are colored as follows: triangles—purple, quadrangles—green, pentagons—yellow, hexagons—grey, heptagons—blue, octagons—orange, nonagons—teal, dodecagons—deep blue.

Type	Tiling	Voronoi Tessellation	
1	*B* + *C* = 180 (*A* + *D* + *E* = 360°)	2 types: 4 5 *S* = 0.693; *ζ* = (1:1)	2 types: 5 6 *S* = 0.693; *ζ* = (1:1)
		2 types: 5 8 *S* = 0.637; *ζ* = (2:1)	
2	*c* = *e* *B* + *D* = 180° (*A* + *C* + *E* = 360°)	2 types: 5 6 *S* = 0.693; *ζ* = (1:1)	2 types: 4 7 *S* = 0.637; *ζ* = (1:2)
		2 types: 4 8 *S* = 0.693; *ζ* = (1:1)	2 types: 5 7 *S* = 0.693; *ζ* = (1:1)
3	*a* = *b*, *d* = *c* + *e* *A* = *C* = *D* = 120° (*B* + *E* = 180°)	3 types: 3 4 6 *S* = 1.011; *ζ* = (2:3:1)	3 types: 3 6 9 *S* = 0.868; *ζ* = (1:4:1)
4	*b* = *c*, *d* = *e*, *B* = 90°, *D* = 90° (*A* + *C* + *E* = 360°)	2 types: 4 7 *S* = 0.637; *ζ* = (1:2)	3 types: 4 6 8 *S* = 0.868; *ζ* = (1:4:1)
5	*a* = *b*, *d* = *e*, *A* = 60° *D* = 120° (*B* + *C* + *E* = 360°)	3 types: 3 6 7 *S* = 0.849; *ζ* = (2:1:6)	3 types: 3 5 6 *S* = 0.849; *ζ* = (2:6:1)
		1 type: 6; *S* = 0 (1)	
6	*a* = *d* = *e*, *b* = *c* *B* + *D* = 180°, 2*B* = *E* (*A* + *C* + *E* = 360°)	3 types: 5 6 7 *S* = 1.099; *ζ* = (1:1:1)	3 types: 3 5 6 *S* = 1.099; *ζ* = (1:1:1)
7	*b* = *c* = *d* = *e* *B* + 2*E* = 360°, 2*C* + *D* = 360° (2*A* + *B* + *D =* 360°)	2 types: 5 8 *S* = 0.637; *ζ* = (1:2)	3 types: 5 6 7 *S* = 1.099; *ζ* = (1:1:1)
8a	*b* = *c = d = e* 2*B* + *C* = 360°, *D* + 2*E* = 360° (2*A* + *C* + *D* = 360°)	2 types: 4 5 *S* = 0.637; *ζ* = (1:2)	3 types: 5 6 7 *S* = 1.099; *ζ* = (1:1:1)
9	*b* = *c* = *d* = *e* 2*A* + *C* = 360°, *D* + 2*E* = 360° (2*B* + *C* + *D* = 360°)	3 types: 5 6 7 *S* = 1.099; *ζ* = (1:1:1)	
10	*a* = *b* = *c* + *e* *A* = 90°, *B* + *E* = 180°, *B* + 2*C* = 360°; (*C* + *D* = 270°)	5 types: 4 5 6 7 8 *S* = 1.516; *ζ* = (2:2:2:4:1)	2 types: 5 12 *S* = 0.305; *ζ* = (10:1)
11	2*a* + *c* = *d* = *e* *A* = 90°, 2*B* + *C* = 360°, *C* + *E* = 180° 2*D* + *E* = 360°	4 types: 4 5 7 8 *S* = 1.386; *ζ* = (1:1:1:1)	4 types: 4 5 6 9 *S* = 1.386; *ζ* = (1:1:1:1)
		2 types: 4 6 *S* = 0.562; *ζ* = (3:1)	
12	2*a* = *d* = *c* + *e* *A* = 90°, 2*B* + *C*= 360°, *C* + *E* = 180° 2*D* + *E* = 360°	2 types: 5 7 *S* = 0.693; *ζ* = (1:1)	3 types: 4 6 8 *S* = 0.950; *ζ* = (1:3:1)
		4 types: 4 5 6 7 *S* = 1.386; *ζ* = (1:1:1:1)	
13	*d* = 2*a* = 2*e* *B* = *E* = 90°, 2*A* + *D* = 360° 2*C* + *D* = 360°	3 types: 5 6 7 *S* = 1.079 (2:3:2)	1 type: 6 *S* = 0 (1)
		3 types: 4 5 7 *S* = 0.956; *ζ* = (1:4:2)	
14	2*a* = 2*c* = *d* = *e* *A* = 90, 2*B* + *C* = 360°, *C* + *E* = 180°; (2*D* + *E* = 360°)	3 types: 5 6 7 *S* = 0.868; *ζ* = (1:4:1)	
15	*a* = *c* = *e*, *b* = 2*a* *A* = *D* = 105°, *B* = 60°, *C* = 13°5 (*E* = 90°)	3 types: 4 5 6 *S* = 1.055; *ζ* = (1:2:2)	
